# Improved Veterinary Service, United States Army1Publication No. 15, American Veterinary Medical Association, Committee on Army Legislation.

**Published:** 1901-01

**Authors:** 


### SOCIETY PROCEEDINGS.

### IMPROVED VETERINARY SERVICE, UNITED STATES ARMY.[1]

We have the honor to submit to the Honorable Senators and Members of the House of Representatives of the United States this copy of a report on the anomalous status of the legislation for an improved veterinary service in the United States Army, and respectfully request their personal consideration of the facts and their favorable action for such a service when such can be taken.

Very respectfully,

D. E. Salmon, Chief of Bureau of Animal Industry, Chairman.
R. S. Huidekoper, Secretary.
A. W. Clement, Baltimore, Md.
Wm. Herbert Lowe, Paterson, New Jersey.
Leonard Pearson, State Veterinarian of Pennsylvania, University of Pennsylvania, Philadelphia.

*Committee on Army Legislation,*
*American Veterinary Medical Association.*

### Preliminary Report of the Committee on Army Legislation of the American Veterinary Medical Association on the Anomalous Status of the Legislation for an Improved Veterinary Service in the United States Army.

We have met with delay in securing an improved veterinary service in the United States Army.

Congress has passed the Army bill and leaves the veterinarian where he was before—without rank, without authority or status over the farrier and enlisted man, and without means of using his best ability for the betterment of the service.

The work of our committee has extended over a number of years.

### History.

Since the days of General Sheridan, when active and promising work was progressing, supported by his intelligent interest, only unpromising and perfunctory efforts were made each session of Congress, consisting of the introduction of bills in the House of Representatives, which died on reaching the Military Committee.

[1] In the third session of the Fifty-fifth Congress the "Hull bill" (H. R. 11022) proposed the rank of second lieutenant for two veterinarians for each regiment of cavalry. In the Senate bill, which superseded the Hull bill, a bill for increasing the efficiency of the army (S. 5578), the Military Committee of the Senate struck out the word "rank" and allowed only the "pay and allowances" of a second lieutentant.

[2] At this period, February, 1899, our committee brought to the atten-

[1] Publication No. 15, American Veterinary Medical Association, Committee on Army Legislation.

tion of the President, William McKinley, the facts: that in England and all European countries there exists an efficient organized veterinary corps in their armies and that the United States Army is absolutely deficient in any such service; that enormous losses had devolved upon our army from glanders and other preventable diseases from want of such a service; that in all other countries the inspection of animal food for the soldiers is made by veterinary officers, who are the educated experts for such service, etc.

The President, Mr. McKinley, referred our representative to the Secretary of War, Mr. Alger. Mr. Alger considered the proposition, said that he wished to see the President before taking action, and made an appointment for the next day. He then indorsed in writing an amendment for the establishment of a Veterinary Corps, which had been prepared by Senator Kenney, of Delaware, and by our committee, and which was offered by Senator Kenney to the Army bill then pending.

On March 2d, as the vote was called in the Senate, upon this amendment Senator Vest obtained the floor for a long speech, and a few hours after the provisional Army bill was adopted without the amendment being acted upon. (See *Congressional Record*, March 2, 1899.)

[3] In June, 1899, the secretary of our committee was given an audience by the President, Mr. McKinley, who gave his approval for renewal of the legislation in the next session of Congress.

In December, 1899, our secretary again called upon the President, related the work of the summer, and was told by the President he wished for the success of it.

During the summer of 1899 our committee collected from Europe the detailed rules and regulations which govern the administration of the veterinary service in her armies, and much additional information of collateral value; submitted to a number of army officers of experience in cavalry, artillery, and transportation service a draft of a proposed service and requested their opinions as to the desirability of such a service.

[4] Answers were received from Major-Generals Wesley Merritt, John R. Brooke, James H. Wilson; Brigadier-Generals J. C. Breckenridge, Wallace F. Randolph, Charles King; General James A. Beaver, ex-Governor of Pennsylvania; Colonels O. L. Hein, Commandant of Cadets at West Point; J. G. C. Lee, Assistant Quartermaster-General, D. D. Wheeler, Deputy Quartermaster-General, and Major John W. Pullman, late Chief Quartermaster, Porto Rico.

These letters were published in pamphlet form and appeared in the *Congressional Record*, May 4, 1899.

[5] In December, 1899, our secretary was given an interview with the Secretary of War, Mr. Root, and, after relating the previous history, presented to him a draft of a proposed veterinary service and accompanying printed data and information, and requested consideration of them. The Secretary of War was gracious in his manner, received the papers, and stated that he would refer them to the General commanding the army and to the Quartermaster-General.

Early in January, 1900, learning that the papers had not been referred to the General commanding or to the Quartermaster-General, our secretary called twice at the War Department and failed to see the Secretary of War, but saw the Adjutant-General, General Corbin, who at each visit rang for a clerk and instructed that the papers should be sent to the Quartermaster-General.

[6] This proposition and the papers were never referred either to the Commanding General or to the Quartermaster-General.

After waiting a week our secretary again called and obtained an interview with the Secretary of War, who said that needs in the medical department and in other departments were so great that he could not consider our proposition.

Senator Kenney then called upon the Secretary of War and received the same answer.

Our committee then carefully considered a form of organization which would make no violent change from existing conditions, but which would provide for a gradual improvement from the present chaotic, unharmonious, inefficient service of civilian veterinary employés into an organized, efficient body.

As the uniform method of the veterinary service in all other armies in the world showed a consensus of opinion that the corps formation is the proper one, we adopted that, on a very small scale, to be gradual in organization; we made it sufficient to be the commencement of what would be an efficient service for the army, but not so large as to become a disturbing factor.

We invite a careful study of this proposed amendment, which, offering slight, gradual promotion, would induce the best class of young, educated veterinarians to enter the service, and would stimulate their best endeavors for the future.

It is as follows:

Senator Kenney's amendment to S. 4300.

Section 14, as S. 4300, passed the Senate May 4, 1900.

Section 20, as S. 4300, passed the House December 6, 1900.

Which are absolutely identical in wording.

[7] That the Veterinary Corps shall consist of:

A chief veterinarian, with the rank, pay, and allowances of a colonel, United States Army.

An assistant chief veterinarian, with the rank, pay, and allowances of a major, United States Army, to be promoted in 1905, after competitive satisfactory examination, from the grade of veterinarian and captain.

Four veterinarians, with the rank, pay, and allowances of a captain of cavalry, to be promoted in 1903, after competitive satisfactory examination, from the grade of assistant veterinarian and first lieutentant.

Ten assistant veterinarians, with the rank, pay, and allowances of a first lieutentant of cavalry, to be promoted, after satisfactory examination, from the grade of assistant veterinarian and second lieutenant after one year's sevice in this grade.

Twenty assistant veterinarians, with the rank, pay, and allowances of a second lieutentant of cavalry, to be appointed after satisfactory examination: *Provided*, That these twenty positions shall include the veterinarians, first class, provided for in the act of March 2, 1899, who have passed satisfactory examinations, and also the six veterinarians, second class, who are now employed in the army under said act of March 2, 1899.

All rules and regulations governing the Veterinary Corps shall be made by the Secretary of War, and the chief veterinarian shall report directly to that officer.

For pay of officers of the Veterinary Corps, $33,500.

[8] Having a consensus of approval and indorsement from an immense majority of all the army officers whom we consulted as to the necessity for such a service, although some varied slightly as to the details in regard to its organization; and having, notwithstanding our request for its consideration, failed to obtain an expression from the War Department as to what alterations and modifications would be acceptable to it, we followed what the study of the veterinary service in other countries had proved to be the most practical and efficient.[1]

At a meeting at which the General commanding, Senator Shoup, Senator Kenney, the Hon. Mr. Hull, Chairman of the Military Committee of the House of Representatives, and others were present, the proposed draft was discussed. The Commanding General, General Miles, stated that he approved it. The Hon. Mr. Hull stated that if the Senate would pass the amendment he would take care of it in his Committee and that the House would pass it.

Senator Kenney then offered it as an amendment to the Army Appropriation bill. The amendment was not considered in the Military Committee of the Senate, and was ruled out on the floor of the Senate on the parliamentary ground of "new legislation."

[9] Senator Kenney again offered the amendment to the several Army bills then pending in the Senate and finally fixed on the Bill for the Efficiency of the Army, S. 4300, as the proper place to bring it up.

The Military Committee of the Senate refused to give our representatives a hearing upon the subject.

[10] Senator Shoup, a member of the Military Committee, in our behalf, requested the Committee to summon the Commanding General, General Miles, and the Quartermaster-General, General Ludington, to express their views upon the subject. His request was refused.

On May 4th Senator Kenney brought the amendment upon the floor of the Senate, and was aided in its advocacy by Senators Wolcott, Gallinger, and Stewart.

Senator Proctor, of the Military Committee, said:

Mr. Proctor: Mr. President, in regard to the reference of this question, I will state that it is not customary for the Committee on Military Affairs to consult all the officers of the Department.[2] There is a regular channel. Bills are referred to the Secretary, and he calls on such officers as have to deal with the subject for their reports. When this bill was before the Committee it was referred in the usual course to the War Department, and I will read the indorsement of the Secretary:

War Department, March 26, 1900.

Respectfully returned to the Committee on Military Affairs, United States Senate, calling attention to the foregoing indorsement.

The foregoing indorsement was that of the Adjutant-General, of course, and was largely quotations from the law, showing that the veterinarians were increased in importance by the act of March 2, 1899. The Adjutant-General says:

The act of March 2, 1899, "for increasing the efficiency of the Army of the United States, and for other purposes," provides (section 2) for two veterinarians in each cavalry regiment, one with the pay and allowances of a second lieutentant of cavalry and one with pay at the rate of $75 per

[1] See 5 and 6. [2] See 6.

month and the allowances of a sergeant-major, and provides "that the veterinarian appointed to the first grade shall not be so appointed until he shall have passed an examination, to be prescribed by the Secretary of War, as to his physical, moral, and professional qualifications;" and "*Provided further*, That the veterinarians now in service who do not pass such competitive examination shall be eligible to the positions of the second class under such rules as are now prescribed by the regulations."

Upon full consideration of the subject at the time it was believed that this provision would provide for efficient veterinarian service for the cavalry regiments.

Now, the Secretary of War says:

The army is urgently in need of more artillery, more staff officers, and more line officers than it now has, and of more soldiers than it will have after June 30, 1901, unless the fighting force of the permanent establishment shall be largely increased by Congress. No bill of this kind ought to pass until these matters of real importance have been provided for. If there is to be any increase, as there should be, it ought to be in the directions indicated. No such rank as is provided for by this bill ought under any circumstances to be given to the Veterinary Corps. If the country is willing to pay for more colonels and majors and captains, the money should be expended upon the fighting force of the army to furnish opportunity for promotion to the hundreds of gallant fellows who have been enduring hardship and facing death on the battlefield.

Nor do I consider that a separate Veterinary Corps is desirable. There are already too many separate organizations reporting to the Secretary of War.

That, I believe, everybody will admit is a truism.

One of the evils of our organization is that of multiple command; that in every geographical or technical organization there are so many officers who are not commanded by the commander, but report directly to Washington.

This provides that the chief veterinarian shall report directly to the Secretary of War.

The result is that it is very difficult to fix responsibility as to the condition of anything, and there is a weakened sense of responsibility everywhere. The Secretary of War ought not to be responsible for the condition of the horses of a cavalry regiment; the colonel of the regiment should be held responsible for them, and the duties of veterinarians should be performed by men who are under his orders, reporting to him, and not by members of a corps reporting separately to the Secretary of War. The bill is distinctly a step in the wrong direction, and I hope it will not receive favorable consideration. Due provision is made for veterinary service in the bill referred to by the Adjutant-General in the first indorsement hereon, to which I beg to invite the attention of the Committee.

ELIHU ROOT,
*Secretary of War.*

(*Congressional Record*, May 4, 1900.)

[11] He also said:

Mr. Proctor: I will say to the Senator that there has been an entire change since the Civil War. Then they had about the pay of a sergeant. Now those of the first class have the pay of a commissioned officer, a first lieutentant.—*Congressional Record*, May 4, 1900.

Note the use of made of "a commissioned officer, a first lieutenant," and then the paragraph of the act referred to, March 2, 1899, as follows:

"Of the veterinarians provided for in this act one shall have the pay and allowances of a second lieutenant of cavalry and one with the pay of seventy-five dollars per month and the allowances of a sergeant-major:

*Provided*, That the veterinarian appointed to the first grade shall not be so appointed until he shall have passed an examination, to be prescribed by the Secretary of War, as to his mental, moral, and professional qualifications: *Provided further*, That the veterinarians now in the service who do not pass such competitive examination shall be eligible to the positions of the second class under such rules as are now prescribed by the regulations. The regimental sergeant-major and the regimental quartermaster-sergeant provided for in this section shall have the pay and allowances of an ordnance sergeant."

(To be continued.)

